# Hypermethylation of the PRKCZ Gene in Type 2 Diabetes Mellitus

**DOI:** 10.1155/2013/721493

**Published:** 2013-03-16

**Authors:** Li Zou, Shirong Yan, Xueping Guan, Yunjun Pan, Xin Qu

**Affiliations:** Clinical Laboratory of Renmin Hospital, The Third Affiliated Hospital of Hubei University of Medicine, 39 Chaoyang Road, Shiyan, Hubei Province 442000, China

## Abstract

*Objectives*. To study the correlation between the methylation of protein kinase C epsilon zeta (PRKCZ) gene promoters and type 2 diabetes mellitus (T2DM). *Methods*. The case-control method was implemented in 272 unrelated to one another cases in Shiyan People's Hospital. Of those, 152 were diagnosed as T2DM cases, and the other 120 cases were healthy individuals visiting the hospital for a physical examination. The subjects were first divided into two groups: the T2DM group and the normal control (NC) group. Next, methylated DNA immunoprecipitation chip (MeDIP-chip) was used for detection. Bisulfite sequencing PCR (BSP) and gene sequencing were then performed to detect and analyze the correlation between PRKCZ gene promoter methylation and T2DM. Finally, Western blotting was applied to determine the serum level of PRKCZ. The data were then analyzed with the statistics analyzing software SPSS 17.0. *Results*. In contrast with cases in NC, T2DM patients showed a high level of methylation, with 7 of 9 CpG sites were shown to be methylated, whereas, in the control group, only one CpG site was found to be methylated. The methylated CpG sites for the two groups showed marked differences (*P* < 0.01). Additionally, the level of PRKCZ was decreased in T2DM subjects, and the difference between the two groups was statistically significant (*P* < 0.05). *Discussion*. This study suggests that the PRKCZ gene is the hypermethylated gene of T2DM and the hypermethylation PRKCZ gene may be involved in the pathogenesis of T2DM.

## 1. Introduction

Type 2 diabetes mellitus (T2DM) is a hyperglycemic endocrine and metabolic disorder caused by insulin resistance and pancreatic *β*-cell dysfunction. It is a complicated, multifaceted disease induced by the combined actions of both genetic and environmental factors [1]. With rapid changes in lifestyle, such as overnutrition and lack of exercise, and aging of the population, the prevalence of diabetes for all age groups worldwide estimated to be 2.8% in 2000 is projected to increase to 4.4% by 2030 [2]. Recent human epidemiological data show over 92 million diabetes patients in China, 90% of which suffer from T2DM [3].

As a polygenic hereditary disease, T2DM has the following characteristics. First, there is usually more than one gene involved in the onset and development of T2DM with each of the genes exerting different functions and intrinsic interactions. Second, T2DM susceptibility genes vary within the population and individuals. A group of people without diabetes might carry the disease susceptibility genes but with a tiny amount. Third, each susceptibility gene plays a role in various metabolic processes of T2DM. Fourth, environmental factors also play an important role in the pathogenesis of T2DM, which suggests that diabetes cases not only demonstrate erratic gene patterns but also show defects in gene expression and post-gene-expressive modification [1]. Additional information is required to fully understand the etiology of T2DM.

In recent years, there has been significant research on the relationship between protein kinase C*ζ* isoforms (protein kinase C epsilon zeta, PRKCZ, or PKC*ζ*) and diabetes [4]. These studies have shown that PRKCZ is a significant contributing factor to T2DM as it is involved in the pathogenesis of T2DM by participating in the impulse transmission of insulin, which is closely related to the function of the pancreas in excreting insulin [5]. The incidence of T2DM involves many factors, such as heredity, environment, and diet. In addition to genetic variations, epigenetics has become the focus of many studies. Thus, it is important to study the correlation between methylation of the PRKCZ gene promoter and the pathogenesis and treatment of T2DM.

## 2. Materials and Methods

### 2.1. Patient Samples

The T2MD group: utilizing the 1999 WHO diagnostic standard, 152 patients, 63 males and 89 females, were diagnosed with T2DM. The cases were excluded from other types of diabetes or endocrine disorders based on clinical examination and family history.

The normal control (NC) group: the control group consisted of 120 healthy unrelated to one another individuals, 55 males and 65 females, with a fasting blood glucose (FBG) <5.6 mmol/L and a postprandial blood glucose (PBG) <7.7 mmol/L.

All the above cases are voluntary.

### 2.2. Research Methods and Procedures

#### 2.2.1. The Peripheral Blood Leukocyte Genomic DNA of the T2DM and NC Patients Was Extracted Using the NaI Method

#### 2.2.2. Analysis by Methylated DNA Immunoprecipitation Chip (MeDIP-Chip) and Identification of the Candidate Gene

Analysis by genome-wide methylation chip was conducted on the extracted DNA (completed by Capital Bio-corporation Co., Ltd.). The entire chromosomal gene sequences of the T2DM and the NC groups were analyzed by MeDIP; the analysis included 22532 gene promoters, fragments from upstream −2.44 Kb to downstream 610 bp, and methylation detection of 27728 CpG islands. Based on the chip analysis results, the PRKCZ gene, the susceptibility gene of T2DM, was identified as the candidate gene as well as the object of study for this research.

#### 2.2.3. Genomic DNA Methylation Was Modified by Sodium Hydrogen Sulfite, Purified, and Recycled

#### 2.2.4. Polymerase Chain Reaction (PCR) and Sequencing Were Conducted

The bisulfite sequencing PCR (BSP) primer was designed according to the CpG islands of the PRKCZ gene promoter using Meth Primer online software and was synthesized by Shanghai Sangon Biological Technology Services Co. Ltd. A 204-bp target fragment was amplified in 35 PCR cycles and sequenced by Shanghai Invitrogen Biotechnology Co. Ltd. Primer sequence:

 Forward primer, 5′-TTTTTGTTTAGGTTGGAGTGTAGTG-3′
 Reverse primer, 5′-CCACTTAAAATCAAAAATTTAAAACC-3′.

#### 2.2.5. PRKCZ Protein Levels in the Peripheral Blood Were Detected by Western Blotting

The serum from the T2DM and NC groups was collected. Western blotting was performed according to the manufacturer's instructions. Serum of T2DM and normal control were collected, and the serum proteins with SDS PAGE electrophoresis were separated; electrophoresis result was shifted to nitrocellulose membrane (NC); NC membrane was soaked the first anti-(1 : 500) of rabbit anti-human PRKCZ antibody which was directly diluted with 5% nonfat dry milk, shaked in room temperature 4 hours or 4°C slow foster overnight, and washed membrane three times with TBST. Added HRP secondary antibody (l : 1000), slowly shaked at room temperature for 2 hours, washed the membrane with TBST four times, each time 8 min. All the experiments were performed in triplicate.

## 3. Results

### 3.1. Screening by NimbleGen MeDIP-Chip

**Figure 1:**
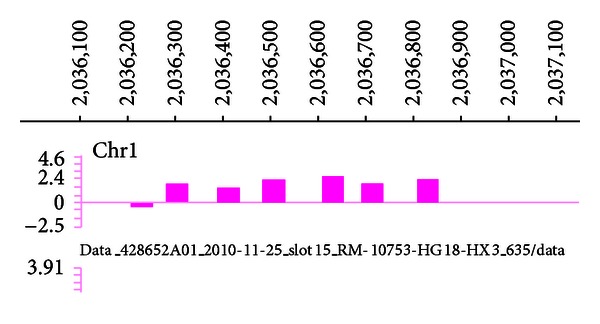
Chromosome 1 promoter region, ■ illustrating detection probe sites of the chip.

The relevant hypermethylated, disease-causing gene of T2DM was determined using MeDIP. The hypermethylated gene was found to be located on Chromosome 1:2036289-2036725. This gene had a Log Ratio of 0.215 and a Peak SCORE of 2.21; Log-Ratio is the value of the probe and Peak score refers to the significance of methylation (Figure 1). The PRKCZ gene promoter is situated at this same site; therefore, the PRKCZ gene promoter was hypothesized to be the hypermethylated gene in T2DM patients. In contrast, the PRKCZ gene promoter showed no obvious methylation in the NC group.

### 3.2. The Methylation Level of the PRKCZ Gene Promoter Is Increased in T2DM Patients

A CpG island-intensive area of 204 bp, a GC content >50%, and an Obs/Exp value >0.6 were found, and this fragment was chosen as the target fragment of BSP. Target fragments of the PRKCZ gene promoter from 272 subjects were amplified by BSP to study the promoter's methylation status. The working principle of BSP is that sodium bisulfite can effectively transform cytosine (C) into uracil (U) in a single strand of DNA. After PCR amplification, uracil (U) in the template is further transformed into thymine (T), while methyl cytosine (^m^C) is not affected by the sodium bisulfite and exists in the unchanged form of deoxidized cytosine (C). Hence, methylation differences between the DNA fragments are transformed into base sequence differences. When the differences have been amplified by BSP, target fragments can then be obtained. Finally, the PCR products are subjected to sequencing and sequence comparisons after modification with sodium hydrogen sulfite, and the methylated CpG sites can be determined.

**Figure 2:**
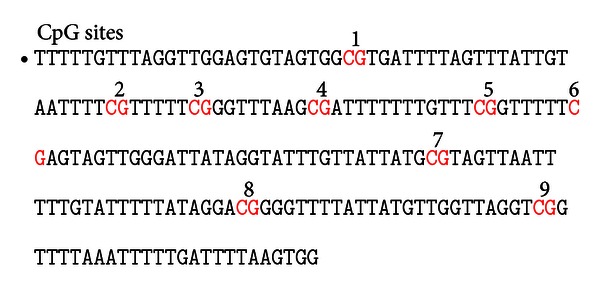
BSP target fragment sequence of PRKCZ gene promoter.

The BSP products from both groups were sent to Shanghai Yingjun Corporation to be sequenced. The data from the two groups were then analyzed to compare the primary gene sequence with the sequence generated after modification by sodium bisulfite. The target fragment of the PRKCZ gene promoter was found to be 204 bp in a length and contain 9 CpG sites (Figure 2). When the detected base sequence of the methylation CpG site remained in the state of C, the site was methylated. After comparing the differences between the base sequences, the degree of methylation could be calculated by ^m^C/CpG.

The comparison between the base sequences in the T2DM and NC groups showed that seven CpG sites were methylated in the T2DM group (7/9 × 100% = 77.7%). CpG sites 2, 3, 6, and 7 showed a high probability of methylation in T2DM patients, whereas only one CpG site (CpG Site 4) was methylated in the NC patients (1/9 × 100% = 11.1%) (Table 1). This is a low level of methylation.

### 3.3. PRKCZ Protein Decreased in the Peripheral Blood Serum in T2DM

The protein expression levels of PRKCZ in the serum of the T2DM and NC groups were analyzed by Western blotting. The expression level of PRKCZ in T2DM patients decreased significantly when compared with the level in the NC group (*P* < 0.05), suggesting that the PRKCZ gene plays a major role in the initiation of T2DM.

## 4. Discussion

### 4.1. Methylation and T2DM

DNA methylation is one of the important molecular mechanisms in epigenetics. It plays an essential role in the maintenance of chromosome structure, X chromosome inactivation, and the expression of genomic imprinting and tumor disease [6]. DNA methylation occurs when a DNA methytransferase (DNMIT) catalyzes the conversion of cytosine into 5-methyl cytosine with s-adenosylmethionine acting as a methyl donor [7, 8]. The modification occurs mainly at the cytosine residue in the two-nucleotide CpG islands. Some research [9] has already shown that DNA methylation is one of the pathogenic factors of T2DM, and it is assumed that DNA methylation plays a role in the initiation and progression of T2DM. PRKCZ is also an important regulatory molecule that affects the signaling pathway of insulin through epigenetic changes. In T2DM patients, there is a disruption of the glucose transport stimulated by insulin, which regulates the transport of blood glucose through interaction with the insulin substrate receptor protein (ISR-1/2) [10], and PRKCZ participates in the occurrence and development of T2DM through insulin signaling pathway mechanisms. Recent studies have identified PRKCZ as a susceptibility gene of T2DM using human genome scanning and fine localization.

### 4.2. Detection of the Entire Genomic Chip in the T2DM Group

The hypermethylated genes of T2DM patients were determined in this study through high-flux screening of the methylated chip. After screening and analysis, PRKCZ was chosen as the target gene for the study. There was no indication of PRKCZ methylation in the normal control group.

### 4.3. Elevation of PRKCZ Gene Promoter Methylation in the T2DM Group

Our findings demonstrate that methylation can be observed in peripheral blood leukocyte DNA from both the T2DM and normal control groups but that the prevalence of methylation of the PRKCZ promoter in peripheral blood leukocytes is significantly higher in the T2DM group (77%) than in the normal control group (7%) (*χ* ^2^ = 36.42, *P* < 0.01).

The fact that methylation has also been detected in the normal control group might indicate that gene methylation not only exists in the development of T2DM but is also an early event of T2DM. Based on previous studies, it was generally hypothesized that the hypermethylation of DNA was correlated with gene silencing, whereas low methylation or demethylation was generally related to gene activation.

Currently, a collection of research [11, 12] indicates that the variation in epigenetics plays a vital role in the initiation and progression of T2DM. Research by Fujiki et al. [13] revealed that high methylation of peroxisome proliferator-activated receptor gamma (PPAR-*γ*) inhibited the gene expression of T2DM, which was regulated by the methylation of its promoter. Research by Ling et al. [14] reported the involvement of methylated peroxisome proliferator activated receptor gamma, coactivator 1, alpha (PPARGCIA) promoter in the development of T2DM. The research showed that the methylation of PPARGCIA promoter in patients with T2DM was twice as much as that in the normal group with the mRNA expression of reduced by 39% and the secretion of islet declined by 34%, which contributed to the reduction of the gene expression, functional disorder of islet and the declined secretion of islet. The methylation of PPARGCIA was closely correlated with T2DM. The expression of PPARGCIA displayed a positive correlation with the secretion of human insulin stimulated by glucose. The above-mentioned studies demonstrate how epigenetic mechanisms might regulate gene expression, such as insulin secretion in human beings.

Moreover, the research suggests that hypermethylation of the glucose transporter protein 2 (GLUT2) promoter suppresses its gene expression and thus leads to the reduced consumption of glucose [15]. Expression of the insulin gene is closely related to the level of methylation at its promoter [16]. These studies indicate that DNA methylation correlates with gene silencing and highly consistent with our study in PRKCZ gene.

In our study, the PRKCZ gene promoter in T2DM patients was hypermethylated while PRKCZ protein expression level in the serum was decreased, suggesting a negative correlation between PRKCZ gene expression and methylation level of the CpG islands. The following inferences may be made from our study. Hypermethylation of the PRKCZ genetic promoter may lead to the reduction of PRKCZ gene expression, which in turn induces the incidence of T2DM via insulin signaling pathways. Alteration of gene expression by DNA methylation may be explained in the following ways. First, methylation leads to structural changes in the genome, such as changes in repetitive sequences and thus suppresses the expression of certain genes [17]. Second, methylation may affect chromosomal structure by shrinking the nucleosome and denying access to the transcription factor, thus leading to instability of the chromosomal structure [18].

Sequencing results demonstrated that the methylation frequency varied by site; the methylation frequencies for the 9 sites were different. The results suggest that the methylation of these sites is probably related to the incidence of T2DM, especially as the frequency of methylation at CpG sites 2, 3, 6, and 7 is equal to or greater than 65%. The findings suggest that the nearby sites might play a role in the regulation of PRKCZ gene expression. This hypothesis must be investigated in future studies.

### 4.4. Prospects of the Study

The study indicates that methylation can serve as a potential diagnostic index of T2DM. As a certain positive rate also occurs in normal tissues, the index could be used as an accessory examination. Higher specificity genes might include some non-detected genes or other genes related to T2DM. Therefore, analysis of these genes could be conducted together with tests of the biopsy samples and peripheral blood of patients, and the occurrence of methylation could be a useful omnidirectional reference for the early diagnosis, prognosis, and clinical therapy of T2DM. It is of great significance in theory and in practice for the primary prevention of T2DM and the screening of a high-risk population.

## Figures and Tables

**Table 1:** The probability of each CpG of methylation.

CpG sites	T2DM group	Normal group	*P *
1	—	—	
2	85%	—	<0.01
3	73%	—	<0.01
4	38%	18%	<0.05
5	15%	—	<0.01
6	75%	—	<0.01
7	65%	—	<0.01
8	35%	—	<0.01
9	—	—	

— indicates that no methylation has been found.

## References

[1] Krupanidhi S, Sedimbi SK, Vaishnav G, Madhukar SS, Sanjeevi CB (2009). Diabetes-role of epigenetics, genetics, and physiological factors. *Journal of Central South University*.

[2] Wild S, Roglic G, Green A, Sicree R, King H (2004). Global prevalence of diabetes: estimates for the year 2000 and projections for 2030. *Diabetes Care*.

[3] Zhou Q, Feng J (2010). The advances of Exendin-4 used for the treatment of type 2 diabetes. *Fudan University Journal of Medical Sciences*.

[4] Zhou L, Wu X, Wang J (2008). Genetic variants in protein kinase C zeta gene and type 2 diabetes risk: a case-control study of a Chinese Han population. *Diabetes/Metabolism Research and Reviews*.

[5] Li YF, Sun HX, Wu GD (2003). Protein kinase C/*ζ* (PRKCZ) gene is associated with type 2 diabetes in Han population of North China and analysis of its haplotypes. *World Journal of Gastroenterology*.

[6] David AC, Thomas J, Danny R (2007). *Epigenetics*.

[7] Pennisi E (2001). Behind the scenes of gene expression. *Science*.

[8] Jones PA, Baylin SB (2002). The fundamental role of epigenetic events in cancer. *Nature Reviews Genetics*.

[9] Maier S, Olek A (2002). Diabetes: a candidate disease for efficient DNA methylation profiling. *Journal of Nutrition*.

[10] Herschkovitz A, Liu YF, Ilan E, Ronen D, Boura-Halfon S, Zick Y (2007). Common inhibitory serine sites phosphorylated by IRS-1 kinases, triggered by insulin and inducers of insulin resistance. *Journal of Biological Chemistry*.

[11] Longnecker DS (2002). Abnormal methyl metabolism in pancreatic toxicity and diabetes. *Journal of Nutrition*.

[12] Gardner RJ, Mackay DJG, Mungall AJ (2000). An imprinted locus associated with transient neonatal diabetes mellitus. *Human Molecular Genetics*.

[13] Fujiki K, Kano F, Shiota K, Murata M (2009). Expression of the peroxisome proliferator activated receptor *γ* gene is repressed by DNA methylation in visceral adipose tissue of mouse models of diabetes. *BMC Biology*.

[14] Ling C, del Guerra S, Lupi R (2008). Epigenetic regulation of PPARGC1A in human type 2 diabetic islets and effect on insulin secretion. *Diabetologia*.

[15] Ban N, Yamada Y, Someya Y (2002). Hepatocyte nuclear factor-1*α* recruits the transcriptional co-activator p300 on the GLUT2 gene promoter. *Diabetes*.

[16] Kuroda A, Rauch TA, Todorov I (2009). Insulin gene expression is regulated by DNA methylation. *PLoS One*.

[17] Tycko B (2000). Epigenetic gene silencing in cancer. *Journal of Clinical Investigation*.

[18] Nguyen CT, Gonzales FA, Jones PA (2001). Altered chromatin structure associated with methylation-induced gene silencing in cancer cells: correlation of accessibility, methylation, MeCP2 binding and acetylation. *Nucleic Acids Research*.

